# Features of Remote Patient Monitoring Systems That Implement Integrated Care: A Perspective Aligned With Current Challenges for Digital Health Technologies: Comment on "Towards A Framework for Implementing Remote Patient Monitoring From an Integrated Care Perspective: A Scoping Review"

**DOI:** 10.34172/ijhpm.8724

**Published:** 2025-03-11

**Authors:** Ana Londral

**Affiliations:** ^1^Value for Health CoLAB, Lisbon, Portugal.; ^2^Comprehensive Health Research Center, Nova Medical School, Nova University of Lisbon, Lisbon, Portugal.; ^3^Department of Physics, Nova School of Science and Technology, NOVA University of Lisbon, Lisbon, Portugal.

**Keywords:** Integrated Care Implementation, Remote Patient Monitoring, Health Technology Design, Digital Health Innovation, Health Service Design, Digital Health Procurement

## Abstract

This commentary elaborates on the model proposed by Miranda et al for implementing remote patient monitoring (RPM) from an integrated care perspective. It stresses the complexity of RPM deployment as a digital health technology (DHT) and discusses essential features that developers and procurement managers should take into consideration in RPM systems to facilitate the implementation of integrated care practices. Furthermore, three major challenges for DHT implementation that align with the proposed RPM-based integrated care model are discussed: (1) the success of DHT in implementing a healthcare strategy requires elements of service innovation that align to the context of care delivery; (2) evidence generation methods influence the adoption of DHT and need an evolutive and multi-stakeholder perspective; (3) governance and policy strategies are crucial since they profoundly influence digital health priorities, investments, and resource allocation within organizations and healthcare systems.

## Introduction

 Remote patient monitoring (RPM) technologies have been developed for several decades, fostering the future prediction of home care with full clinical surveillance. Wireless communication protocols, wearable sensors, internet of things medical devices, intelligent information systems, interoperability, and cybersecurity were topics developed for the last decades and are still emerging in new generations of digital health technologies.^1^

 At this point of history, despite the full stack of technologies available and the knowledge that was generated from public and private investments in research and innovation for the development of eHealth solutions in the last decades, its large scale adoption in society is beyond expectations of companies and innovators that have been developing technology. The concept of “digital determinants of health” is emerging as a crucial view, as highlighted in various significant reports and discussions that link digital transformation in healthcare to broader socioeconomic impacts and strategic health priorities globally.^2^ Though, digital health technology (DHT) advances give us future perspectives for its potential applications to improve healthcare, it is critical to leverage experiences and lessons learned to cumulate knowledge on how to develop and implement DHT that deliver high-value interventions.^3^ Supported on reported experiences and lessons learned, Miranda et al^4^ reviewed conceptual models and real-life initiatives studies and proposed a model for implementing an RPM-Based Integrated Care Initiative.In their paper, the authors formulate relevant elements of an integrated care model that RPM services should follow, organizing these in a 3-tier model, based on a review of conceptual studies and real-life initiatives. Considering the relevance of such framework to help stakeholders in designing integrated care services that rely on RPM, in this commentary I discuss technological features that align with the elements pointed out by Miranda et al.^4^ The aim is to support knowledge for developers and procurement managers on digital tools that can implement those elements. Furthermore, I discuss three challenges related to DHT that are critical to successful implementation in continuum of care and should be considered when applying framework proposed by Miranda et al^4^: (1) RPM success in implementing a care strategy needs elements related to service innovation that align to each context of care delivery. (2) Evidence generation methods influence the adoption of RPM and need to be iterative and have a multi-stakeholder perspective. (3) Strategy and policy are critical since they strongly influence the decisions on implementation priorities and resources in an organization and healthcare system.

## Technological Features of RPM Systems That Implement Integrated Care

 In this section, I suggest concrete features that can guide the development and procurement of the elements proposed in the 3-tier model for implementing an RPM-based integrated care initiative.^4^

###  Tier 1: Elementary Design of an RPM Intervention

 The element *Patient education and self-monitoring promotion* suggests tools that patients can efficiently use to receive information and improve self-care capabilities. Such tools should include simple user interfaces for patients to allow them to monitor their reported data and progression. Also, they can support their engagement and empower them to cooperate for better health outcomes, namely through the use of conversational agents. Bidirectional communication may be used to exchange messages or report data between patients and clinical teams, and have been extensively used for education and monitoring tools in healthcare.^5^ The interaction model of such conversational tools should be customizable to allow adaptation to each according to the context of care. Intelligent chatbots, such as those using generative artificial intelligence (AI), may support patients’ selfcare with reduced need of a clinical team intervention, but models should be submitted to validation to assess its safeness for the target population.^6^

 Caregivers often help patients in reporting measures and can receive education to better support the patient. They should also be considered as potential users of the RPM system, and, in my perspective, are missing in the 3-tier model.

 The element *multidisciplinary workforce* can be implemented through an information system with multiple users, roles and organization affiliations, as RPM allows to connect a network of care teams in different services, in a patient-centric data system. Not only should digital platforms be designed to be accessed by multiple authorized users, but also to connect to other systems and *telemonitoring devices *(element of the model), such as smartphones or wearable medical devices. Compliance with interoperability standards and cybersecurity regulations must be clearly addressed to allow data sharing within the multidisciplinary workforce.

 Implementing an RPM system takes to collect patient-reported data and measures, as also may include the need to collect process and organizational indicators related to patients’ health. As there is no one-fits-all solution, a system that includes customization features for *health indicators *(element of the model) and measurement devices will promote sustainable RPM technologies, in the sense that can be adapted to different contexts of care delivery and patients’ needs.

###  Tier 2: Key Integrated Care Delivery Elements

 Developers and procurement managers should be aware that implementing a new RPM service impacts on health data management, clinical workflows, and other organizational processes. For this reason, when developing or adopting an RPM (or generally, any DHT) to implement a healthcare service, it is essential to implement service design strategies.^7^ To prioritize a *patient-centered implementation* (element of the model), the development and implementation strategies of an RPM should consider the application of participatory methodologies and continuous evidence collection.^8^ The result of applying such methods is new care service models that take a multi-stakeholder perspective into the care pathway, and accommodates multiple perspectives and needs and adapt to the existing healthcare context. The *coordination pivot* (element of the model) can be selected as a project leader to guide a successful implementation in articulation with all the stakeholders involved. As described in Miranda et al, integrated care models need performance indicators that facilitate monitoring and continuous improvement. Taking a value-based healthcare approach, RPM should collect a set of *health outcome measures* (element of the model) that are most relevant to the patients and process outcome measures that impact the organization.^9^ Decision science includes methodologies can be used to gather a set of outcomes, in agreement with the stakeholders involved in the RPM system, that should be possible to collect by the RPM technology (*health indicators* in Tier 1) in the form of eg, questionnaires, internet of things device’s measures, data from IT systems.

###  Tier 3: Added-Value Elements

 From the elements in Tier 3, I highlight the relevance of providing technological features that promote a *culture of collaboration* (element of the model) and coordination among teams and health data-driven shared decisions. In a patient-centered culture, features that promote *shared decision-making* must be considered among clinical teams but also including patients and caregivers who generate and report data and interact with the RPM system. Technological features that allow safe data access by multiple actors should be prioritized since it can facilitate engagement of patients and clinical teams as active decision-makers in an integrated care ecosystem. AI features that support patient profiling and prediction can be also technological features that support the integration of care taking into account the *dynamics of patient trajectories *(element of the model).

## A Service Perspective for RPM Implementation

 As mentioned before in this paper, implementing a DHT may be disruptive as it may change processes, workflows, and demands for new skills and methods from healthcare teams, as also it may be the case of patients. Although the development and procurement activities are often focused on a product (generically, the DHT), it is critical to add a service perspective. A service perspective acknowledges the complexity and dynamism of implementation of a DHT in a specific context. Also, it includes methods to implement it in an organization, in a strategic and accountable manner. Shawn et al^7^ propose a simple heuristic [Tool+Team+Routine] as a method to understand the implications of implementing a DHT for care delivery. The tier “Added-value elements” of the 3-tier implementation model is the most related to this service perspective and must not be underestimated. Developers and procurement managers need to understand that a successful implementation of an RPM that promotes integrated care will depend on the use of methods that acknowledge for service innovation strategies, local adoption specifications, stakeholders’ engagement, or evolving needs.

## Evolving Evidence Generation for Digital Health Technologies

 As Miranda et al^4^ refer “[RPM] technology becomes a facilitator of collecting and sharing information (through ICTs and data centres), enabling coordination (through ICTs and dashboards), and permitting evidence-based actions and continuous improvement (through dashboards and outcome measurement).” In health sciences, “implementation” occurs after an intervention has shown to be effective.^8^ But, for the case of digital health, evidence-generation methods need to consider the broad and dynamic influence of a DHT in healthcare delivery. Developers and procurement managers should be aware that adoption will be an evolving process with real-world evidence as an important factor. Aligned with this vision, the model considers outcomes measures as a key element for integrated care, which allows the collection of evolutionary evidence in real-world. Co-creation methods that involve active users of RPM (patients, caregivers, and clinical teams) in designing the DHT or its local deployment model, defining its benefits and improving evidence of value, taking in consideration cultural aspects and specific needs, are facilitators of success in implementation of care delivery innovation.^9,10^ In this perspective, RPM technologies that include customization features and users’ group profiling will allow progressive evidence generation to multiple stakeholders and different deployment contexts.

## Governance and Policy Strategies Influence Implementation

 As other DHTs, an RPM system, designed to facilitate the implementation of integrated care, necessitates a mission-oriented approach underpinned by an organization or healthcare system’s strategic vision.^9^ Differences in socioeconomic, political and organizational contexts will generate variations in how digital technologies are implemented in the healthcare ecosystem.^3^ Digital health technologies, serving as both tools and engines for the implementation of healthcare policy strategies, must increasingly be recognized as critical determinants of health and trusted as enablers of public good and individual rights.^9^ The establishment of priorities, roadmaps, and national investments in digitally transformed health systems is imperative for the design, development, and iterative implementation of RPM-based digital health interventions^13^ that operationalize the framework proposed by Miranda et al.^4^ For example, a vision of population healthcare that is primarily centered on the specific needs of a target population, irrespective of their geographical proximity^14^ can be a driver for the implementation of an RPM service aligned with integrated care.

 The mission-oriented approach, target population and digital health governance strategy and priorities should be considered in the 3-tier model as major elements that influence the success in implementation of RPM for integrated care.

## Conclusions

 In conclusion, the implementation of RPM systems is inherently complex, necessitating the integration of technology design, service design, and strategy at organizational and policy levels. The work of Miranda et al^4^ is significant in identifying key elements that serve as enablers of successful implementation at both technology and service levels. They refer to current challenges for implementing RPM in a continuum of care, as requiring “coordination and communication between actors and full consideration of involved technology, procedures, and outcome measurement.” This commentary extends the discussion to developers and procurement managers in DHT, aiming to make them aware of technological features that can implement such framework. Furthermore, the paper discusses three challenges related to the implementation of DHT and how the 3-tier model addresses them.

## Ethical issues

 Not applicable.

## Conflicts of interest

 Author declares that she has no conflicts of interest.
